# Comparative efficacy of pharmacological agents on abdominal aortic aneurysm growth rate: a systematic review and network meta-analysis

**DOI:** 10.3389/fphar.2025.1727772

**Published:** 2026-01-15

**Authors:** Cheng Wen, Lilong Li, Bo Wang, Linyang Xie, Huaping Wu

**Affiliations:** 1 Clinical Medical College, North Sichuan Medical College, Nanchong, Sichuan, China; 2 Department of Vascular Surgery, Dazhou Central Hospital, Dazhou, Sichuan, China; 3 School of Medicine and Life Sciences, Chengdu University of Traditional Chinese Medicine, Chengdu, Sichuan, China

**Keywords:** abdominal aortic aneurysm, cohort studies, network meta-analysis, pharmacotherapy, randomized controlled trials

## Abstract

**Background:**

Abdominal aortic aneurysm (AAA) progression lacks proven medications. This study aimed to indirectly compare common drugs’ effects on AAA growth rate using a network meta-analysis (NMA) of randomized controlled trials (RCTs) and cohort studies, assessing the reliability of evidence.

**Methods:**

We systematically searched the Cochrane Library, Embase, Web of Science, and PubMed until 5 June 2025. A Bayesian NMA synthesized direct and indirect evidence on drug effects on AAA growth rate, using standardized mean differences (SMD) with credible intervals (CrI). Cohort study results were analyzed separately.

**Results:**

After screening, 11 RCTs (2,135 subjects) and 13 cohort studies were included. Pooled RCT results showed roxithromycin significantly reduced AAA growth (SMD [95% CrI]: 0.39 [-0.69 to −0.10]). Roxithromycin also demonstrated advantages over amlodipine and doxycycline in indirect comparisons. Propranolol, perindopril, metformin, azithromycin, and ticagrelor showed no significant benefits. Cohort studies linked slower growth to statins and glucose-lowering drugs (insulin, metformin).

**Conclusion:**

Roxithromycin, statins, and metformin show promise for potentially limiting AAA expansion. However, findings are constrained by methodological limitations (study design, sample size), necessitating future validation via high-quality RCTs.

## 1 Introduction

An abdominal aortic aneurysm (AAA) is a chronic degenerative aortic disease characterized pathologically by irreversible dilation of the artery (Kuivaniemi and Elmore, 2012). It is typically defined as a localized enlargement of the abdominal aortic diameter exceeding 50% of the normal diameter, with a clinical threshold often set at >30 mm (Wanhainen et al., 2024). Most unruptured AAAs progress insidiously and lack specific symptoms. As the aneurysm enlarges, patients may experience manifestations of gastrointestinal compression, such as abdominal distension, vomiting, or intestinal obstruction (Moore et al., 2013; Adhikari et al., 2025). Impending rupture or rupture is frequently accompanied by persistent dull pain in the mid-abdomen or lower back, unaffected by posture or activity. Sudden, severe abdominal or back pain, combined with hypotension and a palpable pulsatile abdominal mass, strongly suggests AAA rupture. The mortality rate can reach 85% (Krafcik et al., 2024), with approximately 150,000 to 200,000 global annual deaths attributable to ruptured AAA (Sampson et al., 2014). Consequently, strategies to retard AAA growth and prevent rupture represent critical clinical management goals.

For patients meeting surgical criteria, open repair (OR) and endovascular aneurysm repair (EVAR) are the primary treatment options (Lederle et al., 2019; Buck et al., 2014). However, effective pharmacological interventions are lacking for patients not yet meeting surgical thresholds or deemed unsuitable for surgery, who nevertheless face significant risks of aneurysm progression and rupture. Although certain biomarkers (e.g., fibrinogen, white blood cell count, D-dimer, cardiac troponin T, C-reactive protein) demonstrate associations with AAA, their specificity is insufficient for reliable predictive use (Folsom et al., 2015; Htun and Peter, 2014). Thus, clinical management relies predominantly on serial imaging surveillance of aneurysm size, with maximum diameter considered the most crucial predictor of rupture risk (Owens et al., 2019; Schanzer and Oderich, 2021).

Recent research into AAA pathogenesis suggests that pharmacological therapy may play a role in retarding aneurysm progression. For instance, animal studies by Xiong W et al. and a clinical trial by Lindeman et al. indicated that tetracycline antibiotics can slow AAA development by inhibiting matrix metalloproteinase (MMP) activity and inflammatory responses (Xiong et al., 2012; Lindeman et al., 2009). Furthermore, statins, beta-blockers, angiotensin-converting enzyme inhibitors (ACEIs), angiotensin receptor blockers (ARBs), cyclooxygenase inhibitors, and various anti-inflammatory agents have been reported to potentially influence AAA growth rates (Golledge et al., 2010; Mosorin et al., 2001; Abisi et al., 2008). Certain pharmacological outcomes also reference guidelines from the UK National Institute for Health and Care Excellence (National Institute for Health and Care Excellence: Guidelines, 2020). However, study results are heterogeneous, and systematic comparison and integration are lacking. Currently, there is no conclusive evidence that any specific medication significantly slows AAA growth or reduces rupture risk.

To synthesize the existing evidence regarding the pharmacological inhibition of AAA progression, this investigation employed a systematic review and network meta-analysis (NMA). A key objective was to systematically compare the effects of various common medications on the annual AAA growth rate. The NMA approach allowed for indirect comparisons and ranking of treatments, thereby assessing the reliability of the evidence and providing a quantitative basis for clinical pharmacological intervention in AAA.

## 2 Methods

This NMA adhered to the Preferred Reporting Items for Systematic Reviews and Meta-Analyses (PRISMA) guidelines and its extension for network meta-analyses (Page et al., 2021; Hutton et al., 2015) (Supplementary Table 1). Its protocol was prospectively registered in the International Prospective Register of Systematic Reviews (PROSPERO) (CRD420251070226).

### 2.1 Retrieval strategy

Literature was retrieved from Web of Science, PubMed, the Cochrane Library, and Embase up until 5 June 2025. The search strategy integrated subject headings (MeSH/EMTREE) and free-text terms. Disease-related keywords encompassed ‘abdominal aortic aneurysm’, ‘aortic aneurysm, abdominal’, and ‘AAA’. Intervention-related keywords included ‘drug therapy’, ‘pharmacological treatment’, ‘pharmacotherapy’, ‘pharmacological intervention’, ‘statins’, and ‘fenofibrate’. These terms were combined using Boolean operators (AND, OR) to ensure comprehensive literature retrieval. The Supplementary Tables illustrate specifics.

### 2.2 Eligibility criteria

The inclusion criteria for this NMA were formulated based on the PICOS framework. Population: Individuals diagnosed with AAA, confirmed by computed tomography angiography (CTA) or ultrasound imaging. Intervention: Subjects receiving drug therapy. Comparison: Placebo or other drug treatments. Outcome: AAA’s growth rate or diameter change within the same follow-up period. Study design: Randomized controlled trials (RCTs) or cohort studies (cohort study results were solely for reporting and not included in NMAs). Studies must report data such as mean, standard error (SE), standard deviation (SD), or 95% confidence intervals (CI), enabling direct extraction or calculation of standardized mean difference (SMD).

Exclusion criteria comprised: (i) reviews, commentaries, conference abstracts, case reports, or letters; (ii) studies lacking sufficient data to calculate the SMD and its SE; (iii) research not providing data related to AAA growth or progression; (iv) trials with duplicate or overlapping data.

### 2.3 Literature screening

Two researchers screened the literature independently according to predefined eligibility criteria. Disagreements were resolved through discussion and decision by a third researcher. Initially, the retrieved literature was imported into EndNote to remove duplicates. Subsequent screening of titles, keywords, and abstracts eliminated ineligible literature such as conference papers, guidelines, reviews, animal experiments, and pathological studies. Finally, a full-text review and assessment of the remaining literature determined the final inclusion.

### 2.4 Data extraction

Two researchers independently collected data, including basic information (first author, publication year, study country or region, study type, sample size, participant age, etc.) and outcome indicators (annual growth rate of AAA diameter [cm/y or mm/y], or absolute diameter change [cm or mm] over a specific follow-up period). Due to discrepancies in study duration, measurement instruments, and sample characteristics among various investigations, direct comparisons of the data may be inconclusive. Additionally, while most studies reported annual growth rates (mm/year), some studies (e.g., Baxter et al. (2020)) reported biennial growth rates (mm/2 years). To standardize the data formats across different studies and ensure data integration efficacy, we opted to use the SMD (https://www.cochrane.org/authors/handbooks-and-manuals/handbook/current/chapter-05#, Section-5-3-6). Data reporting annual mean growth speed or absolute diameter changes within the same follow-up period were incorporated into the SMD calculation to ensure the results were comparable.

### 2.5 Quality evaluation

Two reviewers independently appraised the quality of eligible studies employing the National Institutes of Health (NIH) RCT quality evaluation tool (https://www.nhlbi.nih.gov/health-topics/study-quality-assessment-tools). The NIH scale encompasses a broader range of aspects, including research design, implementation details, sample selection, data integrity, and statistical analysis. This tool comprises 14 items, each rated as ‘Yes’, ‘No’, or ‘NR’ based on the rigor of study design and execution. Any discrepancies were adjudicated by a third reviewer. Total scores classified studies as having a high risk of bias (ROB) (poor: 0–5 points), moderate ROB (fair: 6–10 points), or low ROB (good: 11–14 points).

The quality of cohort studies incorporated in the Newcastle-Ottawa Scale (NOS) was also evaluated by two independent reviewers (Ottawa Hospital Research Institute). The NOS covers three main aspects: study selection, comparability, and outcome reporting, including eight items. Except for comparability, which can achieve a maximum of two points, all other items are worth one point, totaling up to nine points. Total scores categorized studies as having a high ROB (poor: 0–4 points), moderate ROB (fair: 5–7 points), or low ROB (good: 8–9 points).

### 2.6 Statistical analysis

According to the guidance provided in Sections 3.2 and 11.3.4 of the Cochrane Handbook (https://www.cochrane.org/authors/handbooks-and-manuals/handbook/current/chapter-03#, Section 3.2; https://www.cochrane.org/authors/handbooks-and-manuals/handbook/current/chapter-11#, Section-11-3-4), RCTs were selected for inclusion in the NMA. This decision was based on the premise that RCTs provide higher-quality evidence than cohort studies. The randomized design effectively mitigates bias, ensuring the reliability of intervention effects. Conversely, while cohort studies can provide evidence from real-world settings, their nonrandomized design makes them susceptible to confounders and biases. Consequently, cohort studies were designated for supplementary analysis rather than inclusion as primary studies in the NMA.

This investigation employed Bayesian NMA to evaluate the impact of various pharmacological interventions on AAA growth rates. By integrating direct and indirect evidence and updating posterior distributions based on prior distributions, the robustness and credibility of our findings were enhanced. All statistical analyses were implemented utilizing R version 4.4.3, with the gemtc package adopted for model construction, which employs Markov Chain Monte Carlo (MCMC) methods for parameter estimation. For continuous outcomes, SMDs and the corresponding SEs were uniformly used for pooled analysis, reporting the pooled SMD and its associated credible interval (CrI). Heterogeneity was evaluated using the I^2^ statistic; a fixed-effect model was applied when I^2^ < 50%, and a random-effect model was adopted when I^2^ ≥ 50%. Results were presented in a league table to illustrate comparative differences among treatments. Surface Under the Cumulative Ranking curve (SUCRA) values were calculated using cumulative ranking curves, with higher values indicating a greater advantage of an intervention in the efficacy ranking.

## 3 Results

### 3.1 Literature screening

**FIGURE 1:**
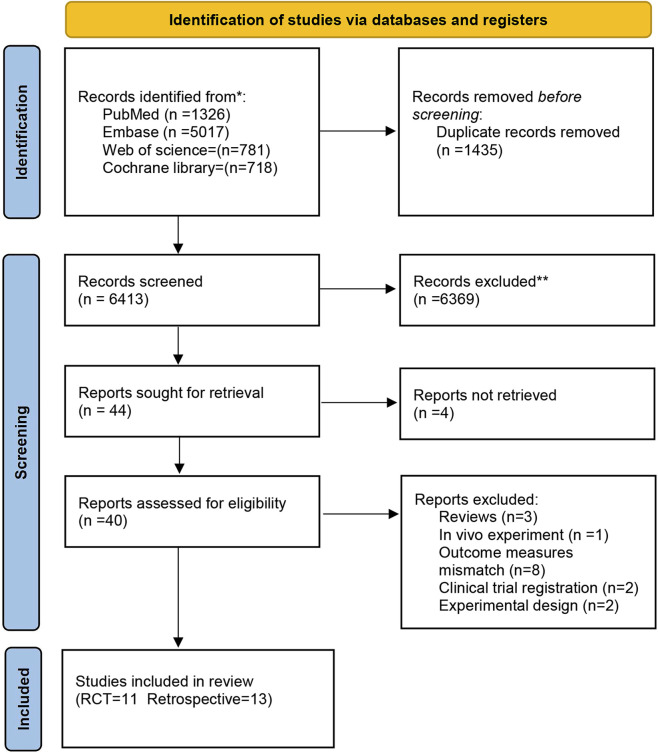
PRISMA flowchart of literature screening.

The initial retrieval yielded 7,842 articles (Supplementary Table 1), comprising 1,326 from PubMed, 5,017 from Embase, 781 from Web of Science, and 718 from Cochrane Library. After deduplication, 6,413 articles proceeded to title and abstract screening. Following the exclusion of 6,369 ineligible articles, 45 articles advanced to full-text evaluation. Finally, four articles were excluded due to unavailability of full text, and 14 were excluded for reasons related to study subjects, intervention, outcome measures, or study design (including one review, one *in vivo* experiment, eight with inconsistent outcome measures, two clinical trial registrations, and two experimental designs). Our NMA encompassed 24 articles, consisting of 11 RCTs and 13 retrospective studies. The literature screening process is depicted in Figure 1.

### 3.2 Basic characteristics

Among the 11 eligible RCTs (Mosorin et al., 2001; Baxter et al., 2020; Bicknell et al., 2016; Meijer et al., 2013; Golledge et al., 2020; Høgh et al., 2009; Eilenberg et al., 2025; Laupacis, 2002; Vammen et al., 2001; Karlsson et al., 2009; Wanhainen et al., 2020), two originated from Denmark, two from Sweden, and the remainder from the United Kingdom, the United States, Finland, the Netherlands, Canada, Australia, and Vienna. The participants generally met the criteria, with studies primarily focusing on individuals with AAAs with baseline diameters ranging from 30 mm to 54 mm. Most subjects were aged 50 or 55 years and older, with an average follow-up period of approximately 1.5–2.5 years. Various medications were compared for their efficacy in slowing AAA growth rates, including antihypertensives (perindopril, amlodipine, telmisartan, propranolol), antibiotics (doxycycline, roxithromycin, azithromycin), hypoglycemics (metformin), and antiplatelet agents (ticagrelor). The primary outcome measure was the annual growth rate of AAA diameter (mm/y) (Table 1).

**TABLE 1:** Characteristics of eligible RCTs.

Study ID	Country/Location	Sample size	Inclusion criteria	Intervening measure	Baseline diameter (mm)	AAA growth mean (SD),mm	Follow-up time (years)	Primary outcome	Study design
Bicknell et al. (2016)	England	79	Aged ≥55 years with an AAA of 3.0–5.4 cm in diameter by internal or external measurement according to ultrasonography and who met the trial eligibility criteria	Placebo	40.6 (6.7)	1.68 (1.78)/y	2	(1) Aneurysm growth rate over 2 years (2) changes in BP; the composite outcome of time taken for the aneurysm to reach the 5.5 cm diameter threshold, referral for elective surgery or AAA rupture; drug intolerance; and drug compliance	RCT
		73		Perindopril	40.5 (6.5)	1.77 (1.71)/y			
		72		Amlodipine	40.3 (6.9)	1.81 (1.70)/y			
Meijer et al. (2013)	Netherland	142	Patients were eligible for the study if they were under surveillance for small AAAs (diameter, 35–50 mm), had Larger AAAs that were unfit for repair, or declined repair	Placebo	43.1 (5.5)	3.30 (2.74)/1.5y	1.5	(1) Aneurysm growth at 18 months (2) growth at 6 and 12 months and the need for elective surgery	RCT
		144		Doxycycline	43.0 (5.5)	4.10 (2.76)/1.5y			
Baxter et al. (2020)	US	125	50 years or older with small (3.5–5.0 cm for men, 3.5–4.5 cm for women) infrarenal aneurysms	Placebo	43.0 (4.0)	3.60 (2.80)/2y	2	Change in abdominal aorticaneurysm maximum transverse diameter measured from CT images at baseline and follow-up at 2 years	RCT
		129		Doxycycline	43.0 (4.0)	3.60 (2.10)/2y			
Golledge et al. (2020)	Australia	101	Participants with 35- to 49-mm AAAs	Placebo	43.1 (5.2)	1.78 (1.77)/y	2	(1) The difference in AAA growth (2) effects on blood pressure (3) computed tomographic (CT)–measured AAA diameter and volume (4)time to AAA-related events (AAA repair or mortality due to AAA rupture) (5) health-related quality of life	RCT
		106		Telmisartan	43.3 (4.8)	1.68 (1.81)/y			
Høgh et al. (2009)	Denmark	42	Male with AAA and residents of viborg county, Denmark, aged 64–73 years	Placebo	37.12 (5.3)	2.52 (2.50)/y	5.27 (1.99)	Expansion rate (mean change in anterior-posterior diameter transformed to annual units) and referral for surgery	RCT
		42		Roxithromycin	38.14 (5.7)	1.61 (1.50)/y			
Eilenberg et al. (2025)	Vienna	28	Non-diabetic patients with an infrarenal AAA with a maximum aortic diameter of 3.0 e 4.9 cm	Placebo	43.57 (6.38)	2.70 (1.93)/1.5y	1.5	Maximum aortic diameter change between baseline and 12 months visit	RCT
		30		Metformin	42.81 (6.38)	2.93 (2.49)/1.5y			
Laupacis. (2002)	Canadian	272	Asymptomatic abdominal aortic aneurysm with a maximum infrarenal diameter between 3.0 and 5.0 cm and no contraindications to propranolol	Placebo	3.92 (0.54)	2.60 (3.10)/y	2.5 (1.1)	(1) The mean annual growth rate as determined by means of ultrasound scanning performed every 6 months (2) Death, surgery, withdrawal from study medication, and quality of life	RCT
		276		Propranolol	3.94 (0.53)	2.20 (2.80)/y			
Vammen et al. (2001)	Denmark	49	Men had an AAA (an infrarenal aortic diameter of 30 mm or more)	Placebo	36.9 (5.1)	2.70 (5.93)2y	2	(1) The mean change in the anterior ± posterior diameter ofthe aneurysm during observation. (2) the proportion of men referred for surgery, the proportion admitted to hospital with cardiovascular disease, the number of deaths and the number of subjects who experienced other adverse events	RCT
		43		Roxithromycin	38.1 (5.7)	1.00 (5.70)/2y			
Karlsson et al. (2009)	Sweden	105	AAA≥35 mm or AAA≥50mm, age ≥81 years, intolerance to macrolides, creatinine learance ≥40 mL/min,ergotamine therapy, including previously diagnosed AAA and newly diagnosed atients meeting inclusion criteria	Placebo	NA	2.17 (1.88)/y	1.5	The expansion rate of the AAA	RCT
		106		Azithromycin	NA	2.34 (1.80)/y			
Wanhainen et al. (2020)	Sweden	70	Patients aged 50–85 years with an AAA between 35 and 49 mm	Placebo	45.1 (5.6)	2.20 (2.35)/y	1	(1) AAA volume growth rate (%) at 12months compared with baseline. (2) AAA-diameter growth rate and intraluminal thrombus (ILT) volume enlargement rate	RCT
		69		Ticagrelor	45.7 (4.7)	2.30 (2.54)/y			
Mosorin et al. (2001)	Finland	15	An AAA diameter perpendicular to the aortic axis of 30 mm or more in size or a ratio of infrarenal to suprarenal aortic diameter of 1.2 or more and a diameter less than 55 mm	Placebo	35.4 (7.4)	3.09 (4.70)/1.5y	1.5	(1) Aneurysm expansion rates. (2) the number of patients who had AAA rupture or repair. (3) C pneumoniae antibody titers. (4) serum concentrations of creactive protein	RCT
		17		Doxycycline	32.4 (8.9)	1.50 (2.43)/1.5y			

The 13 eligible cohort studies (Yaman and Poyraz, 2024; Betancourt-Garcia et al., 2019; Unosson et al., 2021; Karrowni et al., 2011; Schlösser et al., 2008; Periard et al., 2012; Schouten et al., 2006; Gellatly et al., 2024; Itoga et al., 2019; Golledge et al., 2017; Sweeting et al., 2010; Ferguson et al., 2010; Mosori et al., 2008) examined a range of medications, comprising statins, metformin, ACEIs (e.g., enalapril), beta-blockers, and anticoagulants. Baseline diameter ranged from 33.5 mm to 45.34 mm (averaging approximately 38–40 mm). Most follow-up periods concentrated within 2–4 years (Table 2).

**TABLE 2:** Characteristics of eligible cohort studies.

Study ID	Country/Location	Sample size	Intervening measure	Baseline diameter (mm)	AAA growth mean (SD),mm	Follow-up time (years)	Conclusion	Study design
Yaman and Poyraz (2024)	Turkey	234	Antiplatelet	45.24 (6.20)	2.08 (6.58)/3.05y	3.05	Anticoagulant therapy has been associatedwith decreased thrombus diameter and less aneurysmal enlargement compared with antiplatelet therapy. This beneficial effect on the thrombus size and aneurysmal diameter decreased the operational need in patients with anticoagulant therapy	Retrospective
		92	Anticoagulant	45.34 (5.97)	1.31 (6.17)/2.72y	2.72		
Betancourt-Garcia et al. (2019)	Hispanic	68	Statin	38.7 (10.53)	−0.06 (0.54)/Mon	10	This study confirms a difference of AAA physiology between diabetics and nondiabetics in the hispanic community. The observed significant difference in AAA growth rate may be a combination of factors associated with race/ethnicity, prevalence of diabetes mellitus, and low compliance with diabetic control exhibited in the mexican-american population	Retrospective
		68	Beta-blocke		−0.03 (0.54)/Mon			
		10	Insulin		−0.13 (0.97)/Mon			
Unosson et al. (2021)	Sweden	33	Without metformin (T2DM)	37.5 (6.0)	1.6 (1.4)/y	3.2 (1.7)	Metformin prescription is associated with reduced AAA growth rate, possibly mediated by broad anti-inflammatory effects	Retrospective
		65	Metformin (T2DM)	36.5 (5.9)	1.1 (1.1)/y			
Karrowni et a l. (2011)	United States	75	No-statins	41.0 (1.33)	3.38 (2.19)/y	1.04 (0.22)	This is the one of the largest retrospective studies to date demonstrating an association between statin use and decreased growth rate of AAA	Retrospective
		136	Statins	41.0 (2.08)	0.27 (1.50)/y	1.04 (0.27)		
Schlösser et al. (2008)	Netherlands	230	Lipid-lowering drugs users- no users	38.8 (6.8)	−1.20 (8.82)/y	4.0 (2.5)	Lipid-lowering drug treatment and initial AAA diameter appear to be independently associated with lower AAA growth rates	Retrospective
Periard et al. (2012)	Switzerland	44	No statin	40.7 (7.7)	4.37 (3.35)/y	3.21 (2.31)	In this study, patients treated with statin demonstrate a signifi cannot decrease in the annual expansion rate (ER) compared to controls	Retrospective
		20	Statin	39.3 (7.6)	2.91 (2.04)/y			
Schouten et al. (2006)	Netherlands	91	No statin	37.0 (7.0)	3.6 (3.6)/y	3.2	Statins appear to be associated with attenuation of AAA growth, irrespective of other known factors influencing aneurysm growth	Retrospective
		59	Statin	40.0 (8.5)	2.0 (2.3)/y	2.9		
Gellatly et al. (2024)	England	3,670	Inhibitors	37.3	−0.24 (2.16)/y	NA	Metformin with slower abdominal aortic aneurysm growth highlights the importance of the ongoing clinical trials assessing the effectiveness of metformin with regard to the prevention of abdominal aortic aneurysm growth and/or rupture. The association of angiotensin-converting enzyme inhibitors, angiotensin II receptor antagonists, and diuretics with slower abdominal aortic aneurysm growth points to the possibility that optimization of cardiovascular risk management as part of abdominal aortic aneurysm surveillance may have the secondary benefit of also reducing abdominal aortic aneurysm growth rates	Retrospective
			Enalapril		−0.38 (8.50)/y			
			Lisinopril		−0.36 (3.55)/y			
			Perindopril		−0.39 (4.48)/y			
			Ramipril		−0.19 (2.16)/y			
			Aldosterone antagonists		−0.34 (5.56)/y			
			α-Adrenoceptor blockers		−0.17 (2.16)/y			
			Angiotensin ii receptor antagonists		−0.25 (2.47)/y			
			Candesartan		−0.22 (3.71)/y			
			Lrbesartan		−0.11 (8.50)/y			
			Losartan		−0.34 (3.40)/y			
			Calcium-channel blockers		0.03 (2.16)/y			
			Beta-blockers, non-selective		−0.05 (1.55)/y			
			Beta-blockers, selective		0.1 (3.25)/y			
			All diuretics		−0.31 (2.78)/y			
			Bendroflumethiazide		−0.22 (3.25)/y			
			Lndapamide		−0.5 (4.79)/y			
			Loop diuretics		−0.08 (3.71)/y			
			Metformin		−0.38 (3.09)/y			
			Sulfonylureas		−0.34 (5.56)/y			
			Gliptins		−0.32 (6.18)/y			
			Insulin, all classes		−0.17 (7.42)/y			
			Statins		−0.1 (2.47)/y			
			Cholesterol-absorption inhibitors		−0.12 (4.95)/y			
Itoga et al. (2019)	United States	8,342	No-metformin	38.0 (7.1)	1.5 (2.2)/y	4.2 (2.6)	Prescription for metformin was associated with decreased AAA enlargement	Retrospective
		5,492	Metformin		1.2 (1.9)/y			
Golledge et al. (2017)	Australia and new Zealand	99	No-metformin	36.9 (6.3)	1.60 (2.94)/y	3.6 (2.4)	These findings suggest a potential role for metformin in limiting AAA growth	Retrospective
		118	Metformin		1.03 (2.68)/y			
Sweeting et al. (2010)	London	1,532	No-ACEI	NA	2.7 (0.21)	5.3	Patients taking ACE inhibitors have faster aneurysm growth and are in conflict with the observation from a large canadian data-base that aneurysm patients taking ACE inhibitors are less likely to present with aneurysm rupture	Retrospective
		169	ACEI		3.3 (0.21)			
Ferguson et al. (2010)	Australia	303	No statin	33.5 (4.1)	0.78 (0.98)/y	5	The results suggest that statins may have no benefit in reducing AAA progression	Retrospective
		349	Statins	34.9 (3.34)	0.58 (0.90)/y			
Mosorin et al. (2008)	Finland	87	No-statins	39.3 (6.3)	2.6 (2.4)/y	3.6 (2.2)	The use of statins seems to slightly decrease the AAA growth rate and to significantly improve freedom from aneurysm repair and rupture	Retrospective
		34	Statin	38.7 (7.0)	1.9 (1.8)/y			

### 3.3 Quality evaluation

The 11 RCTs were assessed as having good overall quality using the NIH tool. Ten RCTs scored between 11–14 points, earning a ‘Good’ rating. Only Vammen’s study (2001), with a score of 10, was rated ‘Fair’ due to a small sample size and insufficient methodological details. Most studies demonstrated robust practices in randomization methods, allocation concealment, blinding implementation, and outcome measurement. However, some RCTs exhibited limitations. For instance, certain trials mentioned randomization without providing specific procedural descriptions (e.g., Høgh et al. (2009), Mosorin et al. (2001)). Others exhibited minor differences in baseline characteristics (e.g., Eilenberg et al. (2025), Laupacis (2002)). Still others presented inadequate sample sizes or potential confounders (e.g., Karlsson et al. (2009)). Nevertheless, these shortcomings did not significantly compromise the overall quality, indicating a low risk of bias and a high degree of result credibility (Supplementary Table 2).

For the 13 cohort studies, overall assessment was performed using the NOS. The eligible articles generally scored between 8-9 points. Only Periard et al. (2012) and Unosson et al. (2021) presented limitations due to insufficient sample size. Overall, the quality of these articles was high (Supplementary Table 3).

### 3.4 Study findings

Among the 13 cohort studies, evidence predominantly focused on statins for lipid modulation and metformin for glycemic control, constituting the majority of these studies. Multiple studies indicated a correlation between statins and slower annual AAA growth rates. Examples included: 2.0 ± 2.3 vs. 3.6 ± 3.6 mm/y Schouten et al. (2006), 0.27 ± 1.50 vs. 3.38 ± 2.19 mm/y Karrowni et al. (2011), 2.91 ± 2.04 vs. 4.37 ± 3.35 mm/y Periard et al. (2012), and 1.9 ± 1.8 vs. 2.6 ± 2.4 mm/y Mosorin et al. (2008). Within the diabetic subgroup of AAA patients, metformin was associated with slower expansion, as demonstrated by: 1.1 ± 1.1 vs. 1.6 ± 1.4 mm/y Unosson et al. (2021), 1.2 ± 1.9 vs. 1.5 ± 2.2 mm/y Itoga et al. (2019), and 1.03 ± 2.68 vs. 1.60 ± 2.94 mm/y Golledge et al. (2017).

Evidence regarding RAAS inhibitors was mixed. Some studies suggested faster growth with ACEI use; for instance, Sweeting et al. (2010) reported an AAA growth rate of 3.3 ± 0.21 mm/y in the ACEI group versus 2.7 ± 0.21 mm/y in the non-ACEI group, indicating faster aneurysm growth in patients taking ACEIs. Betancourt-Garcia et al. (2019) showed a growth rate of −0.03 ± 0.54 mm/month for beta-blockers compared to no beta-blockers. Yaman and Poyraz (2024) reported AAA growth rates of 2.08 ± 6.58 mm/3.05 years for antiplatelet therapy and 1.31 ± 6.17 mm/2.72 years for anticoagulant therapy.

However, within the 13 eligible RCTs (experimental group vs. control group), only two studies designed by Vammen et al. and Høgh et al., which utilized roxithromycin as an intervention, demonstrated a reduction in AAA growth: 1.00 ± 5.70 vs. 2.70 ± 5.93 mm/2 y and 1.61 ± 1.50 vs. 2.52 ± 2.50 mm/y, respectively. Propranolol also showed a modest effect: 2.20 ± 2.80 vs. 2.60 ± 3.10 mm/y Laupacis (2002). Most other medications, including perindopril, amlodipine, and metformin, did not exhibit significant differences. Notably, Mosorin et al., 2001, which investigated doxycycline, yielded results contrary to those of Baxter (2020) (1.50 ± 2.43 vs. 3.09 ± 4.70 mm/1.5 y versus 4.10 ± 2.76 vs. 3.30 ± 2.74 mm/1.5 y).

### 3.5 Comparative results among drugs

**FIGURE 2:**
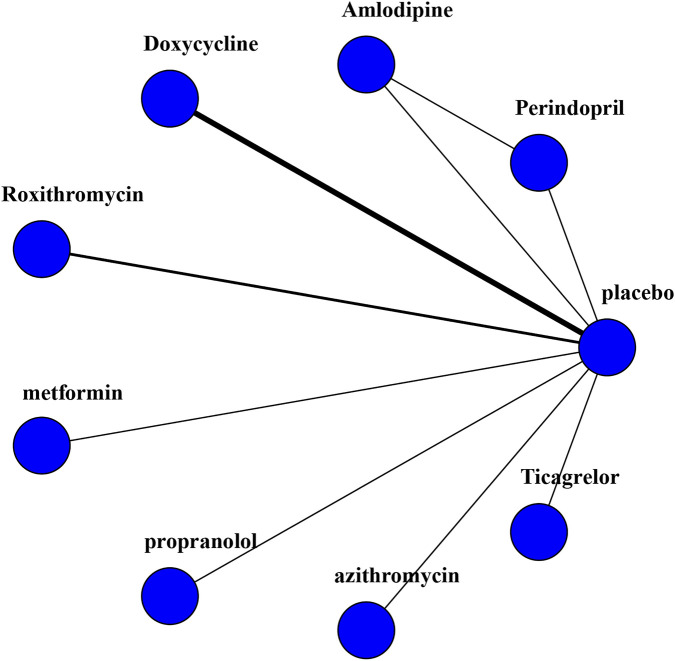
Network plot of the RCT meta-analysis.

A Bayesian NMA was conducted on 11 studies (2,135 participants) involving eight drugs to compare their effects on AAA growth speed (Figure 2). The results indicated that roxithromycin significantly reduced AAA growth velocity (SMD [95% CrI]: 0.39 [-0.69 to −0.10]). It also demonstrated advantages in indirect comparisons with amlodipine (−0.47 [-0.90 to −0.03]) and doxycycline (−0.47 [-0.80 to −0.14]). Perindopril (0.05 [-0.27 to 0.37]), metformin (0.05 [-0.27 to 0.37]), azithromycin (0.09 [-0.18 to 0.36]), and ticagrelor (0.04 [-0.29 to 0.37]) had a limited impact on the annual AAA growth rate (Table 3).

**TABLE 3:** League table from the NMA of RCTs.

Placebo (sucra = 0.53,RANK = 3)	0.05 (−0.27, 0.37)	0.07 (−0.25, 0.39)	0.07 (−0.07, 0.22)	−0.39 (−0.69, −0.1)	0.1 (−0.41, 0.61)	−0.14 (−0.32, 0.04)	0.09 (−0.18, 0.36)	0.04 (−0.29, 0.37)
​	Perindopril (sucra = 0.41,RANK = 5)	0.02 (−0.43, 0.47)	0.02 (−0.33, 0.37)	−0.45 (−0.88, −0.01)	0.04 (−0.56, 0.65)	−0.19 (−0.56, 0.17)	0.04 (−0.38, 0.46)	−0.01 (−0.48, 0.45)
​	​	Amlodipine (sucra = 0.37,RANK = 6)	0 (−0.35, 0.35)	−0.47 (−0.91, −0.03)	0.02 (−0.58, 0.63)	−0.22 (−0.58, 0.15)	0.02 (−0.41, 0.44)	−0.04 (−0.5, 0.43)
​	​	​	Doxycycline (sucra = 0.33,RANK = 9)	−0.47 (−0.8, −0.14)	0.03 (−0.5, 0.55)	−0.21 (−0.44, 0.01)	0.02 (−0.29, 0.33)	−0.03 (−0.39, 0.33)
​	​	​	​	Roxithromycin (sucra = 0.98,RANK = 1)	0.49 (−0.1, 1.08)	0.25 (−0.09, 0.6)	0.48 (0.08, 0.89)	0.43 (−0.01, 0.88)
​	​	​	​	​	Metformin (sucra = 0.36,RANK = 7)	−0.24 (−0.78, 0.3)	−0.01 (−0.58, 0.57)	−0.06 (−0.67, 0.55)
​	​	​	​	​	​	propranolol (sucra = 0.78,RANK = 2)	0.23 (−0.1, 0.56)	0.18 (−0.2, 0.56)
​	​	​	​	​	​	​	Azithromycin (sucra = 0.33,RANK = 8)	−0.05 (−0.48, 0.38)
​	​	​	​	​	​	​	​	Ticagrelor (sucra = 0.43,RANK = 4)

Consistent with the effect estimates, the SUCRA analysis revealed that roxithromycin ranked highest (SUCRA = 0.973), followed by propranolol (0.782) and placebo (0.529) in second and third place, respectively. Ticagrelor (0.429), perindopril (0.404), and amlodipine (0.366) ranked at a moderate level. Azithromycin (0.331) and metformin (0.331) ranked lowest. Nevertheless, it is important to note that SUCRA rankings solely reflect relative probabilities. Differences between certain drugs were not statistically significant in the league table, necessitating cautious interpretation of the rankings.

**FIGURE 3:**
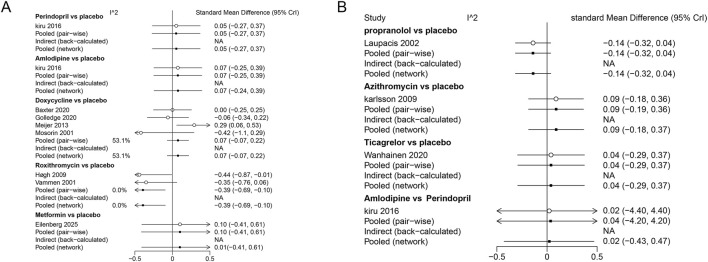
**(A)** Heterogeneity test (part 1/2); **(B)** Heterogeneity test (part 2/2).

Direct comparisons and the pooled analysis from the network model indicated a convergence diagnostic value of 1, suggesting good model fit. Heterogeneity assessment displayed moderate heterogeneity (I^2^ = 52.7%) in the Bayesian comparison model for doxycycline versus placebo. No significant heterogeneity was observed for comparisons between other interventions, with I^2^ values approaching 0% (Figures 3A,B).

## 4 Discussion

Observational cohort studies consistently associated statins and metformin with slower AAA growth. However, inherent limitations like indication bias and residual confounding preclude definitive causal inferences. In contrast, RCT evidence largely failed to confirm that common medications consistently retard AAA progression. Results for amlodipine, doxycycline, and perindopril were predominantly negative, and beta-blockers showed only a minor, statistically uncertain trend. The NMA’s indirect comparisons suggested roxithromycin’s potential superiority. However, this result is derived from two studies with relatively small sample sizes, necessitating cautious interpretation. Additionally, Vammen’s response to his 2002 research (Gibbs, 2002) is noteworthy. Future investigations could focus on evaluating tetracyclines and macrolides with anti-inflammatory activity and no antibacterial properties, regardless of when such agents emerge.

Consistent with previous remarks, this NMA’s observational cohort revealed an association between statins and metformin and a deceleration of AAA growth. However, these findings should be interpreted with caution, as observational studies carry an inherent risk of indication bias and residual confounding factors. For example, metformin’s impact on diabetic patients may be influenced by glycemic control, comorbidity burden, and lifestyle factors. Conversely, biases in statin use could affect their effects. Due to these confounders, results from observational studies may differ from the true effect. We posit that this discrepancy helps explain why metformin yielded incongruent results between observational cohorts and RCTs among the eligible studies. Therefore, metformin’s true effect requires validation in well-designed, multicenter RCTs with adequate follow-up (≥3 years), stratified by diabetic status. Furthermore, no RCTs specifically tested statins for AAA growth retardation. The promising observational signals necessitate prospective randomized evidence to establish causality.

AAA pathogenesis involves the degradation of smooth muscle cells and elastin, which reduces medial density. This is accompanied by inflammatory cell infiltration, neovascularization, and collagen deposition. Inflammation is a key factor in the degeneration of aortic tissue in aneurysms. Plasmin promotes matrix protein breakdown by activating MMPs and transforming growth factor-beta (Schneider et al., 2013; Longo et al., 2002). Animal studies indicate that propranolol may enhance elastin cross-linking and preserve elastin/collagen by increasing lysyl oxidase activity, thereby increasing aortic tensile strength. This delays AAA expansion and reduces the risk of rupture (Brophy et al., 1988; Simpson and Boucek, 1983). The renin-angiotensin system also plays a significant role in AAA development (Hackam et al., 2006). ACEIs and ARBs block this system and its downstream signaling, potentially inhibiting AAA expansion (Kristensen et al., 2015). However, eligible cohort studies have shown mixed results (e.g., Gellatly et al. (2024), Sweeting et al. (2010)). RCTs have provided no supportive evidence. Hyperlipidemia is an independent risk factor. Statins, with their pleiotropic effects (antioxidant, anti-inflammatory, protease inhibition, upregulation of extracellular matrix synthesis), are candidate drugs for slowing AAA growth (Miyake and Morishita, 2009). Previous studies have found that ezetimibe combined with simvastatin reduces aortic wall proteolysis and inflammation (Dawson et al., 2011), while simvastatin slows AAA growth by decreasing MMP-9 concentrations in the aortic wall (Evans et al., 2007.) However, no RCTs have specifically assessed statins for AAA growth reduction, representing a future research direction. Increased thrombus burden and intraluminal thrombus (ILT) correlate with faster AAA growth. ILT promotes neutrophil accumulation, myeloperoxidase release, and localized hypoxia. This alters wall stress distribution, increases extracellular matrix degradation, and reduces wall integrity. Thus, it elevates rupture risk (Parr et al., 2011; Ma et al., 2022; Fontaine et al., 2002). An animal study by Owens et al. (2015) suggests that antiplatelet drugs may reduce rupture risk (Owens et al., 2015). Moran et al. (2017) established an AAA mouse model and found that FXa/FIIa inhibitors limit the severity of aortic aneurysms and atherosclerosis by downregulating PAR-2-mediated Smad 2/3 signaling and MMP-2 expression (Moran et al., 2017). These studies demonstrate the potential of antiplatelet and anticoagulant drugs in slowing AAA progression. Nonetheless, Bicknell et al.'s RCT (Bicknell et al., 2016) found no effect of antiplatelet/anticoagulant therapy on human AAA growth. These findings require validation through larger RCTs. Additionally, interest is growing in mesenchymal stem cell (MSC) therapy for AAA. MSCs target sites of injury or inflammation, potentially modulating T-cell responses (stimulating Tregs, inhibiting CD4+/CD8+ T-cells), preserving elastin, and reducing MMP expression (Schneider et al., 2013). Wang et al. (2018) proposed a study protocol (Wang et al., 2018), but clinical trial results are pending. Advances in nanomaterial-based drug delivery systems (Lu and Hu, 2025) may offer future possibilities for non-surgical AAA management. In summary, roxithromycin, statins, and metformin show potential in slowing AAA growth, but this requires validation in larger studies. Because AAA is often asymptomatic in the early stages, timely detection and intervention for patients with small AAAs or those undergoing regular health screenings could alleviate patient anxiety, reduce long-term healthcare costs, and extend lifespan.

The strengths of this NMA include the inclusion of RCTs across diverse drug classes, such as antibiotics, lipid-lowering drugs, hypoglycemic agents, antiplatelet agents, and anticoagulants, as well as the generally high quality of the eligible studies. Standardized comparisons of multiple interventions and contrasts between RCT and observational findings provide valuable insights. However, one limitation is the restriction of inclusion to studies reporting AAA diameter growth and the exclusion of studies assessing AAA volume, peak wall stress, or MMP-9 changes. These factors could be considered in future analyses. Among the 11 RCTs included in this analysis, the study by Wanhainen et al. (2020) employed two imaging modalities. To minimize the influence of confounders, the guidelines from the United Kingdom National Institute for Health and Care Excellence were referenced, focusing on AAA diameter as measured by ultrasound (National Institute for Health and Care Excellence: Guidelines, 2020). Nevertheless, variations in imaging techniques and follow-up intervals across different studies may result in discrepancies in outcome presentation, contributing to heterogeneity.

## 5 Conclusion

Our synthesis of available evidence indicates that no pharmacotherapy is currently reliably proven to consistently attenuate AAA growth. RCT findings were predominantly negative. Although the NMA suggested a ‘slowing signal’ for roxithromycin, this conclusion is based on small sample sizes and early trials with limited evidence strength and certainty. Therefore, it is not suitable as a basis for clinical recommendations. Consistent observational associations of statins and metformin with slower annual AAA growth rates do not imply causation and require validation through high-quality RCTs. Future large-scale, long-term, multicenter clinical studies are needed to provide robust evidence on the efficacy of these drugs. Additionally, MSC therapy and anticoagulant/antiplatelet agents may offer possibilities for managing AAA without surgery in the future. However, their dosage and administration require further investigation.
