# Supplementary material for: Comparative efficacy of pharmacological agents on abdominal aortic aneurysm growth rate: a systematic review and network meta-analysis
Source: Front Pharmacol. 2026 Jan 15;16:1727772. doi: 10.3389/fphar.2025.1727772 (PMC12851996; doi:10.3389/fphar.2025.1727772)
Supplement: Supplementary file 1 [file Supplementaryfile1.docx]

**Supplementary Table 1:** Search results record

| Pubmed | | |
| --- | --- | --- |
| # | Query | Results |
| 5 | (#1 OR #2) AND (#3 OR #4) | 1329 |
| 4 | "treatment, pharmacological"[Title/Abstract] OR "treatment, drug"[Title/Abstract] OR "therapy, pharmacological"[Title/Abstract] OR "therapy, drug"[Title/Abstract] OR "Therapies, Drug"[Title/Abstract] OR "therapeutic uses"[Title/Abstract] OR "pharmacotreatment"[Title/Abstract] OR "pharmacotherapy"[Title/Abstract] OR "Pharmacotherapies"[Title/Abstract] OR "pharmacological treatment"[Title/Abstract] OR "pharmacological therapy"[Title/Abstract] OR "pharmacological intervention"[Title/Abstract] OR "pharmacologic intervention"[Title/Abstract] OR "pharmaco treatment"[Title/Abstract] OR "pharmaco therapy"[Title/Abstract] OR "pharmaceutical treatment"[Title/Abstract] OR "pharmaceutical therapy"[Title/Abstract] OR "pharmaceutical intervention"[Title/Abstract] OR "pharmaceutic intervention"[Title/Abstract] OR "medicinal treatment"[Title/Abstract] OR "medicinal therapy"[Title/Abstract] OR "medicinal intervention"[Title/Abstract] OR "medication"[Title/Abstract] OR "medicament treatment"[Title/Abstract] OR "medicament therapy"[Title/Abstract] OR "drug treatment"[Title/Abstract] OR "drug therapy"[Title/Abstract] OR "Drug Therapies"[Title/Abstract] OR "Chemotherapy"[Title/Abstract] OR "Chemotherapies"[Title/Abstract] OR "Statins"[Title/Abstract] OR "Fenofibrate"[Title/Abstract] | 1008388 |
| 3 | Drug Therapy[MeSH Terms] | 1561283 |
| 2 | "aortic aneurysm, abdominal"[Title/Abstract] OR "aortic abdominal aneurysm"[Title/Abstract] OR "Aorta Aneurysm, Abdominal"[Title/Abstract] OR "abdominal aortic aneurysm"[Title/Abstract] OR "Abdominal Aorta Aneurysms"[Title/Abstract] OR "abdominal aorta aneurysm rupture"[Title/Abstract] OR "abdominal aorta aneurysm"[Title/Abstract] OR "AAA"[Title/Abstract] | 29074 |
| 1 | abdominal aorta aneurysm[MeSH Terms] | 23982 |

| Embase | | |
| --- | --- | --- |
| # | Query | Results |
| 5 | (#1 OR #2) AND (#3 OR #4) | 5017 |
| 4 | 'treatment, pharmacological':ti,ab,kw OR 'treatment, drug':ti,ab,kw OR 'therapy, pharmacological':ti,ab,kw OR 'therapy, drug':ti,ab,kw OR 'therapies, drug':ti,ab,kw OR 'therapeutic uses':ti,ab,kw OR 'pharmacotreatment':ti,ab,kw OR 'pharmacotherapy':ti,ab,kw OR 'pharmacotherapies':ti,ab,kw OR 'pharmacological treatment':ti,ab,kw OR 'pharmacological therapy':ti,ab,kw OR 'pharmacological intervention':ti,ab,kw OR 'pharmacologic intervention':ti,ab,kw OR 'pharmaco treatment':ti,ab,kw OR 'pharmaco therapy':ti,ab,kw OR 'pharmaceutical treatment':ti,ab,kw OR 'pharmaceutical therapy':ti,ab,kw OR 'pharmaceutical intervention':ti,ab,kw OR 'pharmaceutic intervention':ti,ab,kw OR 'medicinal treatment':ti,ab,kw OR 'medicinal therapy':ti,ab,kw OR 'medicinal intervention':ti,ab,kw OR 'medication':ti,ab,kw OR 'medicament treatment':ti,ab,kw OR 'medicament therapy':ti,ab,kw OR 'drug treatment':ti,ab,kw OR 'drug therapy':ti,ab,kw OR 'drug therapies':ti,ab,kw OR 'chemotherapy':ti,ab,kw OR 'chemotherapies':ti,ab,kw OR 'statins':ti,ab,kw OR 'fenofibrate':ti,ab,kw | 1684072 |
| 3 | 'drug therapy'/exp | 4330715 |
| 2 | 'aortic aneurysm, abdominal':ti,ab,kw OR 'aortic abdominal aneurysm':ti,ab,kw OR 'aorta aneurysm, abdominal':ti,ab,kw OR 'abdominal aortic aneurysm':ti,ab,kw OR 'abdominal aorta aneurysms':ti,ab,kw OR 'abdominal aorta aneurysm rupture':ti,ab,kw OR 'abdominal aorta aneurysm':ti,ab,kw OR 'aaa':ti,ab,kw | 40459 |
| 1 | 'abdominal aortic aneurysm'/exp | 39372 |

| Cochrane Library | | |
| --- | --- | --- |
| # | Query | Results |
| 1 | MeSH descriptor: [Aortic Aneurysm, Abdominal] explode all trees | 881 |
| 2 | ('aortic aneurysm, abdominal' OR 'aortic abdominal aneurysm' OR 'Aorta Aneurysm, Abdominal' OR 'abdominal aortic aneurysm' OR 'Abdominal Aorta Aneurysms' OR 'abdominal aorta aneurysm rupture' OR 'abdominal aorta aneurysm' OR 'AAA'):ti,ab,kw | 2386 |
| 3 | MeSH descriptor: [Drug Therapy] explode all trees | 183271 |
| 4 | ('treatment, pharmacological' OR 'treatment, drug' OR 'therapy, pharmacological' OR 'therapy, drug' OR 'Therapies, Drug' OR 'therapeutic uses' OR 'pharmacotreatment' OR 'pharmacotherapy' OR 'Pharmacotherapies' OR 'pharmacological treatment' OR 'pharmacological therapy' OR 'pharmacological intervention' OR 'pharmacologic intervention' OR 'pharmaco treatment' OR 'pharmaco therapy' OR 'pharmaceutical treatment' OR 'pharmaceutical therapy' OR 'pharmaceutical intervention' OR 'pharmaceutic intervention' OR 'medicinal treatment' OR 'medicinal therapy' OR 'medicinal intervention' OR 'medication' OR 'medicament treatment' OR 'medicament therapy' OR 'drug treatment' OR 'drug therapy' OR 'Drug Therapies' OR 'Chemotherapy' OR 'Chemotherapies' OR 'Statins' OR 'Fenofibrate'):ti,ab,kw | 768639 |
| 5 | (#1 OR #2) AND (#3 OR #4) 726 | 726 |

| Web of Science | |
| --- | --- |
| Query | Results |
| TS=("aortic aneurysm, abdominal" OR "aortic abdominal aneurysm" OR "Aorta Aneurysm, Abdominal" OR "abdominal aortic aneurysm" OR "Abdominal Aorta Aneurysms" OR "abdominal aorta aneurysm rupture" OR "abdominal aorta aneurysm" OR "AAA") | 36630 |
| ALL=("treatment, pharmacological" OR "treatment, drug" OR "therapy, pharmacological" OR "therapy, drug" OR "Therapies, Drug" OR "therapeutic uses" OR "pharmacotreatment" OR "pharmacotherapy" OR "Pharmacotherapies" OR "pharmacological treatment" OR "pharmacological therapy" OR "pharmacological intervention" OR "pharmacologic intervention" OR "pharmaco treatment" OR "pharmaco therapy" OR "pharmaceutical treatment" OR "pharmaceutical therapy" OR "pharmaceutical intervention" OR "pharmaceutic intervention" OR "medicinal treatment" OR "medicinal therapy" OR "medicinal intervention" OR "medication" OR "medicament treatment" OR "medicament therapy" OR "drug treatment" OR "drug therapy" OR "Drug Therapies" OR "Chemotherapy" OR "Chemotherapies" OR "Statins" OR "Fenofibrate") | 1302314 |
| #1 AND #2 | 781 |

**Supplementary Table 2:** NIH quality assessment results for RCTs

| Study ID | 1. Was the study described as randomized, a randomized trial, a randomized clinical trial, or an RCT? | 2. Was the method of randomization adequate (i.e., use of randomly generated assignment)? | 3. Was the treatment allocation concealed (so that assignments could not be predicted)? | 4. Were study participants and providers blinded to treatment group assignment? | 5. Were the people assessing the outcomes blinded to the participants' group assignments? | 6. Were the groups similar at baseline on important characteristics that could affect outcomes (e.g., demographics, risk factors, co-morbid conditions)? | 7. Was the overall drop-out rate from the study at endpoint 20% or lower of the number allocated to treatment? | 8. Was the differential drop-out rate (between treatment groups) at endpoint 15 percentage points or lower? | 9. Was there high adherence to the intervention protocols for each treatment group? | 10. Were other interventions avoided or similar in the groups (e.g., similar background treatments)? | 11. Were outcomes assessed using valid and reliable measures, implemented consistently across all study participants? | 12. Did the authors report that the sample size was sufficiently large to be able to detect a difference in the main outcome between groups with at least 80% power? | 13. Were outcomes reported or subgroups analyzed prespecified (i.e., identified before analyses were conducted)? | 14. Were all randomized participants analyzed in the group to which they were originally assigned, i.e., did they use an intention-to-treat analysis? | score | risk of bias assessment |
| --- | --- | --- | --- | --- | --- | --- | --- | --- | --- | --- | --- | --- | --- | --- | --- | --- |
| Bicknell 2016 | YES | YES | YES | NO | YES | YES | YES | YES | YES | YES | YES | YES | YES | YES | 13 | good |
| Meijer 2013 | YES | YES | YES | YES | YES | YES | NO | YES | YES | YES | YES | YES | YES | NO | 12 | good |
| Baxter 2020 | YES | YES | YES | YES | YES | YES | YES | YES | YES | YES | YES | YES | YES | NO | 13 | good |
| Golledge 2020 | YES | YES | YES | YES | YES | YES | YES | YES | YES | YES | YES | NO | YES | NO | 12 | good |
| Høgh 2009 | YES | YES | YES | YES | YES | YES | YES | YES | YES | YES | YES | NO | YES | YES | 13 | good |
| Eilenberg 2025 | YES | YES | YES | YES | YES | NO | NO | NO | YES | YES | YES | YES | YES | YES | 11 | good |
| Laupacis 2002 | YES | YES | YES | YES | YES | YES | NO | NO | YES | YES | YES | YES | YES | YES | 12 | good |
| Vammen 2001 | YES | YES | YES | YES | YES | YES | NO | NO | YES | NO | YES | NO | YES | YES | 10 | fair |
| Karlsson 2009 | YES | YES | YES | YES | YES | NO | YES | YES | YES | NO | YES | YES | YES | YES | 12 | good |
| Wanhainen 2020 | YES | YES | YES | YES | YES | YES | YES | YES | YES | YES | YES | YES | YES | YES | 14 | good |
| Mosorin 2001 | YES | YES | YES | YES | YES | NO | YES | YES | YES | NO | YES | NO | YES | YES | 11 | good |

**Supplementary Table 3:** NOS quality assessment results for cohort studies

| Study ID | Sample Selection | | | |  |  |  | Assessment of results | | |  |  |
| --- | --- | --- | --- | --- | --- | --- | --- | --- | --- | --- | --- | --- |
|  | Representativeness of exposed cohort | Selection of nonexposed cohort | Ascertainment of exposure | Absence of outcome at start of study |  | Comparability of cohorts |  | Outcome assessment | Length of follow-up | Adequacy of follow-up |  | risk of bias assessment |
| YaMan 2024 | ⭐ | ⭐ | ⭐ | ⭐ |  | ⭐ |  | ⭐ | ⭐ | ⭐ |  | Good |
| Monica 2019 | ⭐ | ⭐ | ⭐ | ⭐ |  | ⭐ |  | ⭐ | ⭐ | ⭐ |  | Good |
| Karrowni 2011 | ⭐ | ⭐ | ⭐ | ⭐ |  | ⭐⭐ |  | ⭐ | ⭐ | ⭐ |  | Good |
| Schlösser 2008 | ⭐ | ⭐ | ⭐ | ⭐ |  | ⭐ |  | ⭐ | ⭐ | ⭐ |  | Good |
| Periard 2012 | ⭐ | ⭐ | ⭐ | ⭐ |  | ⭐ |  | ⭐ | ⭐ | ⭐ |  | Good |
| Schouten 2006 | ⭐ | ⭐ | —— | ⭐ |  | ⭐ |  | ⭐ | ⭐ | ⭐ |  | Good |
| Gellatly 2024 | ⭐ | ⭐ | ⭐ | ⭐ |  | ⭐⭐ |  | ⭐ | ⭐ | ⭐ |  | Good |
| Itoga 2019 | ⭐ | ⭐ | ⭐ | ⭐ |  | ⭐⭐ |  | ⭐ | ⭐ | ⭐ |  | Good |
| Golledge 2017 | ⭐ | ⭐ | ⭐ | ⭐ |  | ⭐⭐ |  | ⭐ | ⭐ | ⭐ |  | Good |
| Sweeting 2010 | ⭐ | ⭐ | ⭐ | ⭐ |  | ⭐⭐ |  | ⭐ | ⭐ | ⭐ |  | Good |
| Ferguson 2010 | ⭐ | ⭐ | ⭐ | ⭐ |  | ⭐ |  | ⭐ | ⭐ | ⭐ |  | Good |
| Mosorin 2008 | ⭐ | ⭐ | ⭐ | ⭐ |  | ⭐⭐ |  | ⭐ | ⭐ | ⭐ |  | Good |
